# *Notes from the Field:* Development of an Enhanced Community-Focused COVID-19 Surveillance Program — Hopi Tribe, June‒July 2020

**DOI:** 10.15585/mmwr.mm6944a6

**Published:** 2020-11-06

**Authors:** Royce Jenkins, Rachel M. Burke, Joyce Hamilton, Kathleen Fazekas, Duane Humeyestewa, Harpriya Kaur, Jocelyn Hirschman, Kay Honanie, Mose Herne, Oren Mayer, Graydon Yatabe, S. Arunmozhi Balajee

**Affiliations:** ^1^Hopi Tribe, Kykotsmovi, Arizona; ^2^CDC COVID-19 Response Team; ^3^Hopi Health Care Center, Polacca, Arizona; ^4^Laboratory Leadership Service, CDC.

The Hopi Tribe, a sovereign nation in northeastern Arizona, includes approximately 7,500 persons within 12 rural villages (*1*). During April 11–June 15, 2020, the Hopi Health Care Center (HHCC, an Indian Health Services facility) reported 136 cases of coronavirus disease 2019 (COVID-19) among Hopi residents; 27 (20%) patients required hospitalization (J Hirschman, MD, CDC, personal communication, June 2020). Contact tracing of Hopi COVID-19 cases identified delayed seeking of care and testing by persons experiencing COVID-19–compatible signs and symptoms*; inconsistent adherence to recommended mitigation measures,^†^ such as mask-wearing and social distancing; and limited knowledge of the roles of testing, isolation, and quarantine procedures^§^ (*2*). Based on these findings, the Hopi Tribe Department of Health and Human Services (DHHS) collaborated with HHCC to develop a community-focused program to enhance COVID-19 surveillance and deliver systematic health communications to the communities. This report describes the surveillance program and findings from two field tests.^¶^

The Hopi Tribe DHHS, HHCC, and CDC collaborated to develop methodology and materials for this surveillance program, which aimed to expand upon the Community Health Representative Program. The Hopi Tribe DHHS administers the Community Health Representative Program, which provides health education and patient follow-up through home visits to patients referred by HHCC. Community health representatives are salaried employees with basic clinical training; each manages a caseload of 30–40 patients in one or two villages. For surveillance field tests, community health representatives visited every household in two villages.** At each household, community health representatives screened each member for COVID-19–like signs and symptoms^††^ and exposures using a standardized form, recommended testing where indicated, and provided education on everyday prevention activities and mitigation of within-household transmission of SARS-CoV-2, the virus that causes COVID-19, using culturally adapted materials.^§§^ Symptomatic or exposed persons were referred for SARS-CoV-2 testing and management at HHCC. Safety provisions for community health representatives included wearing personal protective equipment, conducting interviews outdoors, maintaining a distance of ≥6 feet from interviewees, and limiting close contact with households reporting confirmed COVID-19 cases (i.e., providing education to well household members from a distance of ≥6 feet but not conducting interviews).

Field tests of the surveillance protocol in two smaller villages were conducted on June 24 in Oraibi and on July 16 in Bacabi (estimated populations 100 and 175, respectively). Five two-person teams, each composed of one community health representative and one volunteer (from the village, Hopi Tribe DHHS, or CDC field team), canvassed each village within 5 hours. In the two villages, 101 households were approached, 78 (77%) of which provided basic information on 259 persons (Table); 141 were screened (age range = 1–91 years, median = 50 years). Two persons who reported mild COVID-19–like symptoms (nasal congestion and runny nose) and two possibly exposed persons were referred for testing. Only the exposed persons sought testing; both received negative test results by reverse transcription–polymerase chain reaction (nasopharyngeal swabs were sent to a commercial laboratory for analysis). One mildly symptomatic person did not permanently reside with the family and was lost to follow-up, and one mildly symptomatic person reported that symptoms were attributable to seasonal allergies. Based on interactions, teams reported that residents of the two villages seemed appreciative of the program and of community health representative presence and were receptive to COVID-19 health education.

**TABLE:** Numbers of households reached and residents interviewed, by village, in two field tests of house-to-house COVID-19 surveillance and community education* — Hopi Tribe, June–July 2020

Characteristic	No. (%)		
	Village		Total
	Oraibi^†^	Bacabi^§^	
**Total no. of households approached**	**33**	**68**	**101**
No one home	0 (—)	18 (26)	**18 (18)**
Household declined	1 (3)	4 (6)	**5 (5)**
Household accepted interview	32 (97)	46 (68)	**78 (77)**
**Total no. of residents in interviewed households**	**103**	**156**	**259**
Persons screened for COVID-19–like signs and symptoms^¶^ and exposures	64 (62)	77 (49)	**141 (54)**
Persons declined screening	0 (—)	4 (3)**	**4 (2)**
Persons unavailable for screening	39 (38)	75 (48)	**114 (44)**
Persons referred for testing	4 (6)	0 (—)	**4 (2)**

**Abbreviation:** COVID-19 = coronavirus disease 2019.

* Five two-person teams, each composed of one community health representative and one volunteer (from the village, Hopi Tribe Department of Health and Human Services, or CDC field team), canvassed each village within 5 hours.

^†^ Canvassed on June 24, 2020.

^§^ Canvassed on July 16, 2020.

^¶^ Fever, chills, body aches, fatigue/extreme tiredness, headache, runny nose, nasal congestion, sore throat, new change/loss in smell or taste, cough, shortness of breath, chest pain, vomiting/nausea, diarrhea, and abdominal pain.

** All four were children whose parents declined screening on their behalf.

In this rural, low-resource setting, house-to-house COVID-19 surveillance and education was feasible, as evidenced by the use of 10 staff members to screen 141 persons in <10 hours, and well-accepted, as indicated by a 5% household refusal rate (Table). Data on reasons for which households declined screening and education were not systematically collected, but involvement of community health representatives, who are known and trusted in the communities, likely increased acceptability of the program. Community health representatives identified a need for increased engagement with village leadership to improve identification of nonvacant houses and availability of household members. Public health guidance about COVID-19 prevention and mitigation strategies was shared with households, including recommendations on when to seek testing, how and when to wear masks and practice social distancing, hand hygiene, and proper isolation and quarantine. Given positive feedback on this program from the communities, community health representatives, HHCC, and the Hopi Tribe leadership, each Hopi village was canvassed at least once during July–October 31, 2020, and resources will be sought to expand the program to canvas villages on a more frequent basis. Additional potential modifications to the program include streamlining the household interview and distributing masks. If the program is expanded, it will be evaluated after 1 year of implementation according to predefined indicators for impact on COVID-19 case detection and community knowledge and practices; precise details of this evaluation plan have not yet been finalized.

## Footnotes

* https://www.cdc.gov/coronavirus/2019-ncov/symptoms-testing/symptoms.html.

† https://www.cdc.gov/coronavirus/2019-ncov/downloads/php/open-america/community-mitigation-quicklinks.pdf.

§ https://www.cdc.gov/coronavirus/2019-ncov/if-you-are-sick/index.html.

** These villages were not the site of the outbreak that occurred during May–June 2020 among extended family members.

†† The following signs and symptoms were specifically asked about: fever, chills, body aches, fatigue/extreme tiredness, headache, runny nose, nasal congestion, sore throat, new change/loss in smell or taste, cough, shortness of breath, chest pain, vomiting/nausea, diarrhea, and abdominal pain.

§§ Materials included a laminated booklet with information on how to safely isolate and quarantine in smaller houses that might lack running water; a flyer highlighting important prevention messages such as hand hygiene, mask-wearing, and social distancing; and the Community Health Representative newsletter, which reinforced prevention messages and also provided contact information for resources accessible by tribal members.

## References

[1] Hopi Tribe. The Hopi Tribe: the official website. Kykotsmovi, AZ: Hopi Tribe; 2020. https://www.hopi-nsn.gov/

[2] Hirschman J, Kaur H, Honanie K, A SARS-CoV-2 outbreak illustrating the challenges in limiting the spread of the virus—Hopi Tribe, May–June 2020. MMWR Morb Mortal Wkly Rep 2020;69. 10.15585/mmwr.mm6944a5 PMC7643893 33151922

